# Defining culturally safe primary care for people who use substances: a participatory concept mapping study

**DOI:** 10.1186/s12913-020-05915-x

**Published:** 2020-11-23

**Authors:** Karen Urbanoski, Bernadette Pauly, Dakota Inglis, Fred Cameron, Troy Haddad, Jack Phillips, Paige Phillips, Conor Rosen, Grant Schlotter, Elizabeth Hartney, Bruce Wallace

**Affiliations:** 1 Technology Enterprise Facility, Canadian Institute for Substance Use Research, University of Victoria, PO Box 1700 STN CSC, Victoria, BC V8W 2Y2 Canada; 2 School of Public Health and Social Policy, University of Victoria, PO Box 1700 STN CSC, Victoria, BC V8W 2Y2 Canada; 3 School of Nursing, University of Victoria, PO Box 1700 STN CSC, Victoria, BC V8W 2Y2 Canada; 4 SOLID Outreach Society, 1056 N Park St, Victoria, BC V8T 1C6 Canada; 5 Umbrella Society for Addictions and Mental Health, 415 Dunedin St #8, Victoria, BC V8T 5G8 Canada; 6 Centre for Health Leadership and Research, Royal Roads University, 2005 Sooke Road, Victoria, BC V9B 5Y2 Canada; 7 School of Social Work, University of Victoria, PO Box 1700 STN CSC, Victoria, BC V8W 2Y2 Canada

**Keywords:** Canada, Cultural safety, Substance use, Primary care, Stigma, Concept mapping, Community based participatory research

## Abstract

**Background:**

People who use substances experience high levels of substance-related stigma, both within and outside of health care settings, which can prevent people from help-seeking and contribute further to health inequities. Recognizing and respecting how political, social, economic, and historical conditions influence health and health care, cultural safety, with origins in addressing Indigenous racism, is a potential strategy for mitigating stigma and marginalization in health care. Using a participatory research approach, we applied the concept of cultural safety to develop a model of safe primary care from the perspective of people who use substances.

**Methods:**

People who use or used substances were involved in all phases of the research and led data collection. Study participants (*n* = 75) were 42.5 years old on average; half identified as female and one quarter as Indigenous. All were currently using or had previous experience with substances (alcohol and/or other drugs) and were recruited through two local peer-run support agencies. Concept mapping with hierarchical cluster analysis was used to develop the model of safe primary care, with data collected over three rounds of focus groups.

**Results:**

Participants identified 73 unique statements to complete the focus prompt: “I would feel safe going to the doctor if …” The final model consisted of 8 clusters that cover a wide range of topics, from being treated with respect and not being red-flagged for substance use, to preserving confidentiality, advocacy for good care and systems change, and appropriate accommodations for anxiety and the effects of poverty and criminalization.

**Conclusions:**

Developing a definition of safe care (from the patient perspective) is the necessary first step in creating space for positive interactions and, in turn, improve care processes. This model provides numerous concrete suggestions for providers, as well as serving as starting point for the development of interventions designed to foster system change.

## Background

Over the past few years, many countries have been experiencing large increases in fatal and non-fatal drug overdoses, largely attributable to the increased presence of synthetic opioids in the illicit drug supply [1–4]. In Canada, there were over 15,000 apparent deaths linked to overdoses between 2016 and 2019, with 77% of these fatal overdoses involving fentanyl or fentanyl analogues [5]. Among the lessons being learned as policy makers, service providers, researchers, and the public navigate this crisis is an appreciation of the extent to which the existing system of services for people who use substances falls short of meeting population needs.

Primary care has an important role to play as a key point of entry to the health care system. Many people who use substances, particularly when substance use is combined with structurally produced disadvantages, such as poverty and homelessness, do not have access to primary care [6, 7]. Among an array of patient, provider, and system-level factors, the stigma of substance use is a barrier to timely and effective health care. To inform ongoing efforts to improve the quality of health care for people who use substances, we used the concept of cultural safety to guide an investigation of what constitutes safe, accessible, and acceptable primary care for this population.

### Impact of stigma on help-seeking and health outcomes

The stigma associated with substance use is deeply embedded in health and social systems as a result of current policies that criminalize drug use and neo-liberal beliefs that people who use substances are solely to blame for their problems. Illicit drug use, in particular, is highly stigmatized, especially when coupled with structural disadvantages (e.g., homelessness, colonialism, racism, classism, sex and gender discrimination, poverty) and other marginalized health conditions (e.g., HIV/AIDS, HCV) [8–13]. Socioeconomic disadvantages and associated processes of social exclusion contribute to inadequate access to health care [8, 9, 14–19]. Feelings of not being safe in health care settings, due to fear of judgements and stereotyping, can lead people to delay and avoid seeking the health care they require [13, 20–23].

Studies have shown that many primary care providers including physicians, community health workers, and nurses hold negative and stigmatizing beliefs towards people who use and experience harms related drugs and alcohol [22, 24, 25]. Lack of trust has been identified as a key concern that impacts access to primary care [21, 23, 26]. While harm reduction services (e.g., needle and pipe distribution, supervised consumption sites) are often more trusted as a source of care [27, 28], their capacity to provide primary care is limited by the lack of integration with primary care services. For people who use substances, particularly illicit drugs in combination with structurally produced disadvantages, the overall impact is one of reduced access to primary care in general and lack of safety when accessing primary care.

### Cultural safety

Cultural safety was originally developed by Indigenous nurse scholars to address racism and improve health care practice for Indigenous peoples in New Zealand [29–31]. The Nursing Council of New Zealand has outlined cultural safety as an approach to care that requires health care providers at all organizational levels to 1) reflect on their own, often unconsciously held, attitudes and beliefs about others; 2) examine ways in which history, social relations, and politics continue to shape peoples’ health needs and access and responses to care, and 3) demonstrate flexibility in how they relate with others, especially those who are different from themselves. In the Canadian context, cultural safety is relevant to understanding the impact of historical trauma and ongoing institutionalized racism impacting Indigenous peoples’ health, health care experiences and access to health care [32–34]. It has been proposed as a strategy for mitigating stigma, discrimination, and other forms of marginalization in health care for both Indigenous and non-Indigenous people [35, 36] and has been applied more broadly to other groups experiencing marginalization and differences in power and privilege [13].

Conceptually, cultural safety emphasizes recognition and respect for how political, social, economic, and historical conditions influence and shape health, health care, and health inequities [35, 37, 38]. Cultural safety extends beyond cultural sensitivity and competency, by focusing attention on power imbalances, systemic discrimination, social contexts, and impacts on health inequities [38, 39]. It is defined from the perspective of those receiving care, in terms of what makes them feel safe or unsafe in a health care setting. Because it is defined by care recipients, it challenges care providers to reflect on their own cultural identity, positions of privilege and power, and how these influence (often unconscious) assumptions about health and health care practice. Cultural safety offers a lens through which people’s health can be seen as influenced by their historical, socio-political, and socioeconomic contexts, and accordingly though which health care providers can work towards equity and social justice within their professions [37, 38].

Given the impacts of substance-related stigma and social marginalization on health and health care inequities, cultural safety is a potentially useful concept to guide the study of how to improve care and care experiences for people who use substances. Prior work has examined how negative constructions of illicit drug use (e.g., as an individual failing, criminal activity, or disease) impacted on patients’ experiences and nurses’ abilities to provide culturally safe care in hospital [13]. To date, however, there has been no systematic exploration of the meaning of culturally safe primary care from the perspective of people who use substances. Developing a definition of safe care (from the patient perspective) is the necessary first step in creating space for positive interactions and, in turn, improve care processes.

### Objectives

As part of a broader research agenda focused on developing recommendations to reduce stigma and foster greater inclusion of people who use substances in health care planning and research, we conducted a concept mapping study to investigate how people who use substances understand cultural safety in primary care. The study and its approach were developed by a project team that includes people who use or have used drugs, academic researchers, health planners, and health care providers.

## Methods

This study followed a participatory community-based research approach, which “integrates education and social action [into research] to improve health and reduce health disparities” (p. 312 [40]). People who use or used substances were involved in all phases of the research, and the research process itself had a goal of social action related to reducing stigma in primary care settings. The initial study was conceptualized through a collaboration of people with lived experience, academic researchers, health planners, administrators, and providers. The core research team was composed of community researchers (people who use or used substances) and academic researchers with the remainder of the initial planning team acting as advisors and contributing to knowledge translation. The community researchers were affiliated with two local peer-run organizations that provide support, advocacy, and outreach services to people who use substances and who also experience various forms of structural disadvantages related to poverty and homelessness, racialization, criminalization, and discrimination. The two peer-run organizations operate from different philosophies, with one working from a harm reduction philosophy and the other (at least historically) embracing a more abstinence-based understanding of recovery.

We used concept mapping to develop a model of safe primary care for people who use substances [41, 42]. We chose concept mapping for this study since the concept of culturally safe primary care has not been previously described for this population. The approach involved five steps, detailed below. Data were collected in three rounds of focus groups with people who use or used substances, led by the community researchers with assistance from the academic researchers. Data collection took place from October 2017 to January 2018. Ethical approval for this study was obtained from the institutional Research Ethics Boards at the University of Victoria and the Vancouver Island Health Authority.

### Preparation and participant recruitment

The first step in concept mapping consists of establishing a focused prompt and identifying a plan for recruitment and data collection [42]. The focus prompt refers to a statement or question to be completed or answered by participants in step 2, serving as the basis for the concept to be defined. We developed the focus prompt, recruitment, and data collection strategies in a series of face-to-face research meetings of the core research team. The final focus prompt for mapping the concept of culturally safe primary care was: “I would feel safe going to the doctor if …” Relevant health care settings included doctors’ offices, walk-in clinics, and other community health clinics, but excluded emergency and acute care settings. Prospective participants were people who use or used substances (alcohol and/or other drugs) and who experience structural disadvantages (as described above). The sample was recruited by the community researchers via word-of-mouth through their networks, and attention was given to recruiting people who were diverse in terms of gender, age, and current use of substances (abstinent and non-abstinent). Written informed consent was obtained at the first data collection point, with the assistance of a video recording in which one of the community researchers described the study and meaning of consent.

Participants were 42.5 years old on average (SD = 12.1). Half identified as female and one quarter as Indigenous (i.e., First Nations, Inuit, or Métis) (Table 1). Three quarters had at least some post-secondary education. Most (61.3%) lived in a rented house or apartment, and 73.6% described their housing situation as stable. All participants were people who were actively or had formerly used substances, had experienced substance-related harms or problems, and were known to the two peer-run support agencies involved in the study.

**Table 1:** Sample description (*n* = 62)

Characteristic	n (%)
Gender	
Female	31 (50.00)
Male	30 (48.38)
Other gender identity	1 (1.62)
Racial/Ethnic Background ^a^	
White	50 (80.65)
Indigenous	15 (24.19)
Other	10 (16.13)
Current accommodations	
Rented house/apartment	38 (61.29)
Drug treatment residence	5 (8.06)
Shelter/Refuge	2 (3.23)
Other	17 (27.42)
Stability of Housing	
Yes	45 (72.58)
No/No fixed address/Other	17 (27.42)
Highest level of education completed	
Less than high school	15 (24.19)
Completed high school	13 (20.97)
Post-secondary	34 (54.84)

^a^ Participants could identify more than one category, so the percentages sum to more than 100%

### Brainstorming

In the second step participants are asked to brainstorm as many ideas as possible to complete the focused prompt. This step was also completed during the first round of focus groups. Each focus group was facilitated by at least one community researcher and one academic researcher. A total of 68 participants attended 16 focus groups (3–8 people per group). Participants’ ideas were recorded on a flip chart by the academic researcher and transcribed after the groups. In addition to stipends, food was available at the focus groups for participants. A total of 754 statements were generated, including duplicates generated in different groups. Participants also completed an anonymous questionnaire providing sociodemographic information. After the groups were completed, the core research team reviewed the statements and removed duplicates. Through extensive discussion, similar ideas were collapsed and synthesized to create the final list of statements. The team reached consensus on a final set of 73 statements that represented all of the ideas voiced by participants in the focus groups.

### Sorting

In the second round of focus groups, participants sorted the statements into groups based on conceptual similarity. A total of 56 people participated in this round of focus groups, including 54 of the 68 recruited initially (79.4%) with the addition of 2 new participants. The statements were printed on sets of index cards, which the participants individually sorted into physical piles. They were instructed to create as many piles of cards as they wanted to represent the concept of cultural safety. The number of piles per participant varied from 2 to 20. In these groups, participants were also asked to reflect on whether the 73 statements accurately captured the discussions from the first focus groups, and whether any ideas were missing. No new statements were suggested.

### Analysis

Data were entered for analysis in Concept Systems. The analysis involved generating a 73 × 73 matrix in which the cells contained the number of times participants grouped each pair of statements together. The matrix was analyzed using multi-dimensional scaling (MDS) to produce a 2-dimensional point map of statements. Statements grouped together more often appear closer together on the point map. Goodness of fit between the 2-dimensional configuration and the original input matrix was assessed using a measure of stress. The stress value is a normalized residual variance after accounting for the association between the configuration and input matrices, and thus provides a measure of the extent to which the produced configuration is random or without structure [43, 44]. Values range from 0 to 1, with lower values indicating better fit. After 19 iterations the stress value provided for this study was 0.325, which falls within the recommended range (> 0.39).

We then used hierarchical cluster analysis to identify discrete clusters of statements based on the point map. To decide on the optimal number of clusters, the core research team reviewed the output from analyses producing 4 to 12 clusters of statements in a face-to-face meeting. Starting with the 12-cluster solution, we examined the groups of statements and reflected on participants’ discussions of statement meaning in the focus groups. We worked successively through the cluster maps with n-1 clusters, considering the conceptual meaning of the clusters as they merged, until further merges were felt to result in a loss of meaning and specificity. In this way, consensus was reached for the 8-cluster solution as a comprehensive and meaningful representation of the data.

### Interpretation

In the third and final round of focus groups, study participants were asked to review the 8-cluster solution and cluster map, and to identify the key ideas that were conveyed by each cluster. A total of 48 people participated in this round of focus groups, including 43 of the 68 recruited initially (63.2%) and 5 new participants (75 people participated in the study overall; 72% participated in more than one round of focus groups, and 57.3% participated in all three). In addition to identifying key themes for each cluster, participants also rated each of the 73 statements in terms of importance, on a scale from 1 (not important) to 5 (very important). The statement ratings were entered into Concept Systems, and the mean and standard deviation calculated for individual statements and clusters (i.e., mean and SD for all statements within a cluster).

## Results

**Fig. 1:**
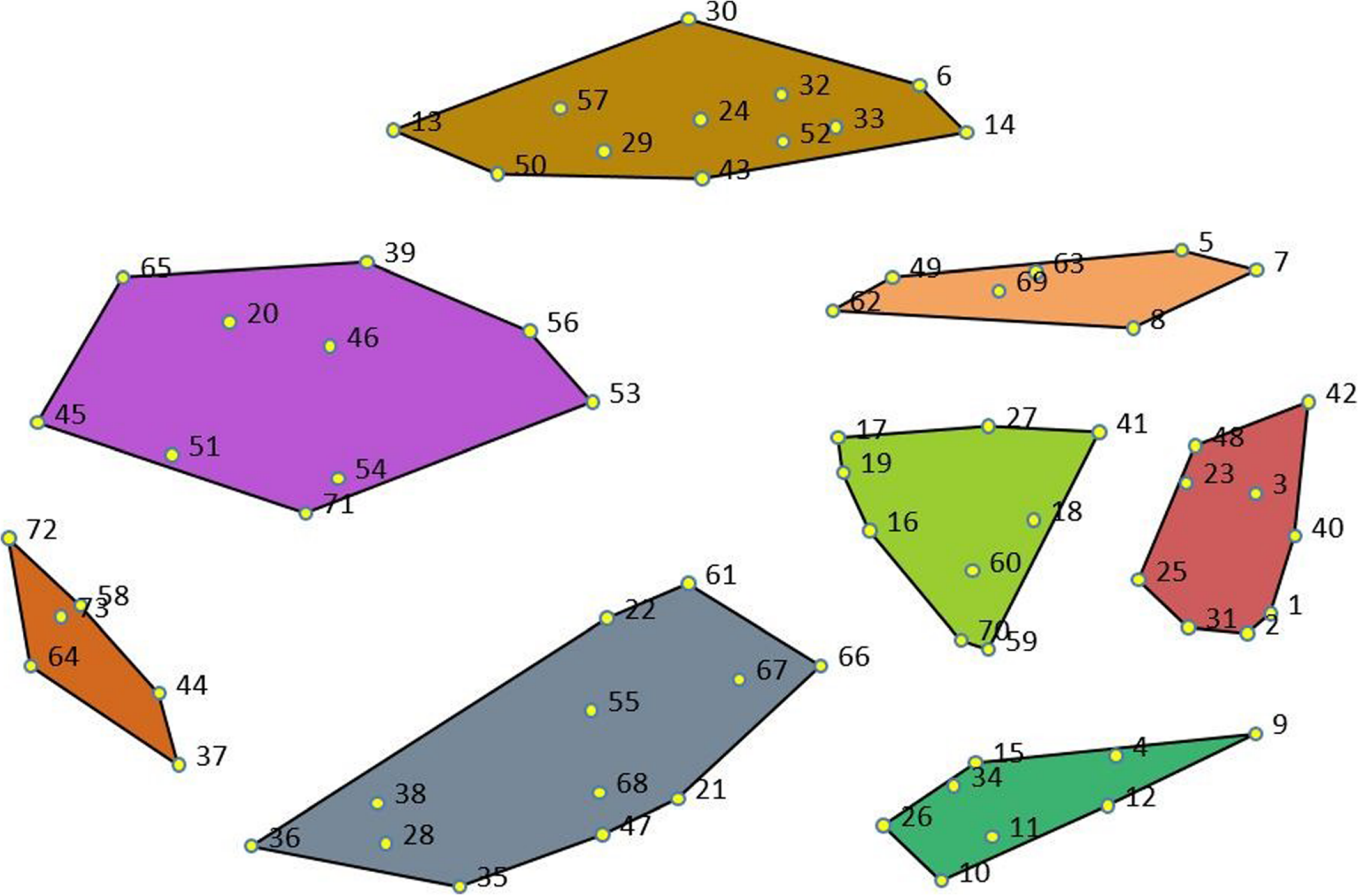
Cluster map^a^. ^a^Numbers correspond to the statements listed in Table 2

Participants drew from both positive and negative experiences to relate their ideas of culturally safe primary care; that is, they recalled examples of both safe and unsafe health care experiences. The 73 statements, grouped by cluster, are shown in Table 2 along with the mean ratings (and SD) of importance. Figure 1 shows the cluster map (the point map with the 8-clusters superimposed).

**Table 2:** Clusters and statements^a^

I would feel safe going to the doctor if …	Rating of importance, mean (SD)^**b**^
**Cluster 1 - Act to prevent stigma: Don’t treat me like crap!**	**4.18 (0.23)**
5. I knew that I wouldn’t be judged or labeled	4.23 (0.95)
7. I wasn’t embarrassed, ashamed, or thought they would be disappointed in me	3.88 (1.04)
8. I was treated like a human being, seen as more than my illness	4.44 (0.65)
49. I knew they wouldn’t make assumptions about me without knowing me	4.30 (0.81)
62. They recognized that some issues are hard to talk about, and provided accommodations to help reduce my anxiety	4.07 (0.98)
63. I wasn’t seen as threatening	3.93 (0.96)
69. I knew they would respect boundaries	4.43 (0.86)
**Cluster 2 - Hey, I’m human. Treat me right!**	**4.29 (0.26)**
1. They were caring, compassionate, and patient	4.46 (0.58)
2. I knew that they were looking out for me and my health	4.54 (0.68)
3. They treated me fairly, with respect and dignity	4.52 (0.77)
23. They trusted my knowledge of my own health and what works for me, and didn’t talk down to me	4.15 (0.93)
25. They would tell me what they are doing, what my diagnosis is, and what is happening with my care.	4.47 (0.55)
31. They were open to letting me ask for a second opinion, or at least admit it if they don’t know something	4.20 (0.65)
40. They look at me and make eye-contact.	4.04 (0.91)
42. They don’t make irrelevant comments or ask irrelevant questions, but just dealt with the problem at hand.	3.79 (0.88)
48. I knew they would listen to me, take me seriously, and let me ask questions	4.43 (0.69)
**Cluster 3 - Uphold professional standards**	**4.24 (0.19)**
16. They would understand and adequately treat my pain	4.37 (0.71)
17. They would get my medications right, not cut me off or over-medicate me	4.44 (0.80)
18. They gave me information about medications, side effects, and interactions	4.25 (0.84)
19. They had up-to-date knowledge on drug interactions and didn’t prescribe me medications that interact with others that I am taking	4.52 (0.74)
27. I had a say in my treatment planning and decisions	4.28 (0.83)
41. They are professional and impartial, and don’t act surprised when I tell them things	4.13 (0.80)
59. My priorities are my doctor’s priorities	4.22 (0.94)
60. I didn’t have to beg for tests	4.00 (0.94)
70. They would ask me more questions	3.98 (0.92)
**Cluster 4 - Do you care about me?**	**3.99 (0.17)**
4. The visit wasn’t rushed, and we could talk about more than one problem	3.94 (0.89)
9. I knew that I would get the help that I need, even if that means they have to put in a bit of extra effort	4.23 (0.79)
10. I could see the same doctor and didn’t have to repeat my story each time	3.92 (1.01)
11. The doctor and I trust each other well and have a rapport	4.19 (0.82)
12. They are personable and have a good bedside manner. It’s not just “what’s your name” and “what’s your problem,” but “how are you” and “how is your day?”	3.79 (1.00)
15. I was offered a variety of options, not just medications	4.13 (0.79)
26. Care was holistic (addressed mind, body and spirit) and helped get to the root of underlying problems	3.98 (0.87)
34. They would follow-up or check in with me about my care.	3.77 (1.00)
**Cluster 5 - Maintain my confidentiality in a welcoming and comfortable environment**	**3.87 (0.43)**
21. They didn’t ask the reason for my visit in the waiting room, or talk openly about other patients	4.13 (1.02)
22. They wouldn’t diagnose me without knowing me. Don’t misdiagnose me.	4.42 (0.74)
28. I didn’t have to sit in a waiting room for a long time. Being able to book an appointment (online or by phone) is helpful.	3.74 (0.92)
35. The office was comfortable and not institutional	3.28 (1.14)
36. The waiting room wasn’t too small or packed	3.26 (1.13)
38. I could speak with my doctor on the phone	3.37 (1.22)
47. The receptionist and office staff were friendly and welcoming	3.80 (0.99)
55. I could see my file/medical record	3.76 (1.26)
61. I knew that my providers communicated with each other (so bad interactions between medications are avoided, my doctor knows the results from consultations, etc.)	4.15 (0.84)
66. I knew they would record my information accurately	4.57 (0.62)
67. I felt that we were accountable to each other	4.16 (0.68)
68. They were open to feedback or criticism, or there was an easy process for making a complaint	3.76 (0.99)
**Cluster 6 - Be a champion for advocacy**	**3.37 (0.62)**
37. Services are easy to access and in safe and non-triggering locations	3.53 (1.18)
44. The doctor or nurse was the same gender as me	2.58 (1.32)
58. A family member, friend or advocate could come with me	3.63 (1.34)
64. The doctor was part of my family circle. This is how it works in Indigenous communities.	2.73 (1.29)
72. I knew I was covered by insurance	4.24 (0.77)
73. They had a safe, discrete location for giving me my pain medication	3.48 (1.11)
**Cluster 7 - Acknowledge and accommodate patient needs and circumstances**	**3.81 (0.54)**
20. I knew that my information would be kept confidential and private	4.54 (0.71)
39. It was a multi-service clinic just for people who use drugs or have addictions	3.43 (1.14)
45. It were easier to get contraception	2.89 (1.29)
46. They were not sexually inappropriate	4.26 (0.85)
51. I knew they were a good doctor (if they were highly recommended, or if my friends were using them)	3.76 (0.92)
53. They knew that I only come to the doctor when I am really sick	3.39 (1.14)
54. They would understand if I’m late or if I miss an appointment, and wouldn’t charge me	3.40 (1.19)
56. I knew they wouldn’t force care on me that I don’t want, or do things without my consent	4.48 (0.69)
65. I knew they wouldn’t take my kids away	3.84 (1.07)
71. They understood my circumstances and lack of money.	4.15 (0.84)
**Cluster 8 - Don’t red flag me: Recognize addiction as a health issue**	**4.16 (0.29)**
6. I knew that I wouldn’t be treated badly or lectured about my drug use	4.44 (0.77)
13. I wasn’t blacklisted, red-flagged, or refused care	4.35 (0.84)
14. I knew they wouldn’t assume I am drug-seeking or trying to scam the system	4.23 (0.75)
24. They were open to harm reduction	4.02 (0.71)
29. I knew that I could get access to mental health care, even though I use drugs	4.43 (0.65)
30. They know about and can help me to access services and resources for people who use drugs	4.22 (0.63)
32. They have up to date knowledge about drug use and addiction, and understand the connection with mental health	4.38 (0.80)
33. They know where we are coming from and that drug use isn’t all about motivation	3.85 (0.93)
43. I knew that addiction would be treated like any other health problem	4.16 (0.85)
50. I know I wouldn’t be in trouble with the law or treated like a criminal	4.20 (0.81)
52. They are accepting of drug use, so that I could speak openly about it	4.20 (0.83)
57. The doctor can relate to my experiences of drug use	3.43 (1.19)

^a^ Statement numbers appear in Fig. 1

^b^ Ratings vary from 1 (not important) to 5 (very important)

*Cluster 1: Act to prevent stigma: Don’t treat me like crap!* The seven statements in this cluster (light orange on the cluster map) defined aspects of safety that had to do with not feeling judged, stigmatized, or treated poorly because of substance use. They are fairly closely grouped together, indicating a high degree of conceptual similarity within the cluster. Statements reflect both external stigma (being judged by others) and internalized stigma (feeling embarrassed or ashamed). This cluster reveals how keenly the stigma of substance use is felt by people who use substances, who fear being labeled as threatening and being shamed or embarrassed in primary care settings.

*Cluster 2: Hey I’m human. Treat me right!* Closely related to the above is another tightly grouped cluster of nine statements (red) that reflects the importance people placed on being treated fairly and with compassion from the first point onward. People highlighted the importance of being trusted as a patient (in terms of being taken seriously and having their knowledge of their own health recognized). They wanted to know that they could ask questions and that their provider would provide explanations about their health and health issues. These were understood as elements of safety that came with knowing you would be respected as a person and treated “right” (like a human). Although closely aligned with Cluster 1 (as indicated by the close positioning of the two clusters on the cluster map), the statements in Cluster 2 speak generally to being treated with respect and dignity, while those in Cluster 1 more explicitly address fears of judgment, labeling, and stigma.

*Cluster 3: Uphold professional standards.* Cluster 3 (light green) contains nine tightly connected statements that concern professional competencies, particularly as they relate to substance use, addiction, medications, and related health issues (especially pain management). A central element in this cluster revolved around how important it was that providers recognize people’s claims of pain as credible and legitimate, and that pain be adequately managed. Closely related to this was being included in care planning and decision-making. Together, these were seen as key elements of professional competency that translated into feeling safe at the doctor’s office.

*Cluster 4: Do you care about me?* Located below these three clusters on the map, Cluster 4 (dark green) contained eight tightly connected statements that speak to the safety that is felt when care is personalized, when there is continuity of care, rapport, and trust between the physician and patient. People noted challenges related to not being able to see the same physician at each visit and feeling rushed during appointments. Attention to these details was seen as demonstrating care, which in turn contributed to people’s sense of safety.

*Cluster 5: Maintain my confidentiality in a welcoming and comfortable environment.* Next to these was a larger cluster of 12 statements (grey) that were not as tightly connected as in previous clusters. Of particular concern were the waiting room environment and general clinic practices and protocols. People described safe primary care environments as welcoming and comfortable with a chief priority being maintenance of confidentiality by all in the clinic setting. For example, needing to provide a reason for their visit in a crowded waiting room that could be overheard by others, and overhearing providers and/or office staff having conversations about other patients constituted negative and unsafe experiences. People feared that they would be being misdiagnosed, that their information would not be recorded correctly, or that physicians would not communicate with each other about their health conditions. Concrete suggestions for creating safety included accommodations around these issues included not being asked questions in the waiting room, being able to speak with the doctor on the phone or being able to see their own medical records were provided by participants.

*Cluster 6: Be a champion for advocacy.* Somewhat separated off from the other clusters was a small cluster of six tightly connected statements (dark orange) that address the accessibility of services and the possible supports needed to reduce anxiety and improve perceived safety in accessing care. Examples included allowing people to bring an advocate or friend with them to appointments, having doctors of the same gender as the patient, providing care in discrete and accessible locations, and having system supports in place (e.g., avoiding situations in which people are not covered by insurance). It was noted that, in Indigenous communities, doctors are part of one’s family circle. These issues were all seen to reflect a possible role for primary care providers as advocates for people who use substances, both clinically and systemically to ensure culturally safe primary care.

*Cluster 7: Acknowledge and accommodate my needs and circumstances.* Above these was another larger (more dispersed) cluster of 10 statements (purple) that highlight socioeconomic and other circumstances that affect the ability of people who use substances to navigate health and social service systems. The statements reflect the importance of primary care providers understanding and accommodating patient circumstances and assisting in mitigating negative consequences. Perceptions of safety were linked to provider recognition that people living on low incomes may not have the resources to execute plans of care, or that life circumstances and demands make following plans of care difficult. Examples of concrete suggestions for improving perceived safety included forgiving charges for missed appointments, not punishing people for missing appointments or labelling them as non-compliant, explicitly asking to ensure patient consent for procedures, and recognizing and accommodating fears of losing child custody or being criminalized if drug use is disclosed. This cluster indicates the need for a public health approach to primary care that recognizes the structural (economic, gendered, and socio-legal) conditions that impact people’s lives and involves accommodations to ensure they can access services.

*Cluster 8: Don’t red flag me: Recognize addiction as a health issue.* The final cluster (brown), at the top of the point map, contains 12 statements that link safety to physician views that addiction is a legitimate health issue, not a criminal behavior. This included aspects of physician knowledge (about addiction and available services and supports), acceptance of the reality of drug use, and openness to harm reduction strategies. People indicated that in order to feel safe accessing primary care they did not want to be red-flagged by the health care system as a drug user for fear this would impact their ability to receive ongoing physical and mental health care.

On the 5-point scale (ranging from 1 to 5), mean ratings of statement importance ranged from 2.58 to 4.57. The top 5 ratings (all over 4.5) were for statements in clusters “Maintain my confidentiality in a welcoming and comfortable environment,” “Hey, I’m human. Treat me right!” “Acknowledge and accommodate patient needs and circumstances,” and “Live up to professional standards.” Only three statements were rated below 3.00 (the mid-point) in importance, including two from “Be a champion for advocacy” cluster and one from “Maintain my confidentiality in a welcoming and comfortable environment.” All clusters had statements rated above 3.00 and there was no obvious pattern to average ratings across the clusters, suggesting relatively equal contributions to the overall concept of culturally safe primary care.

## Discussion

This study provides a structured conceptualization of culturally safe primary care for people who use substances. Culturally safe primary care for people who use substances recognizes is respectful care, provided with explicit attention to confidentiality, professional standards, and the impacts of structural conditions (including criminalization and poverty). The eight themes that we identified comprise a comprehensive overview that can be used as a starting point for the development of interventions designed to foster system change, and for systematic evaluation of the facilitators and barriers to implementing quality improvement initiatives. Participants were readily able to describe their understandings of safe primary care, covering a wide range of topics from features of the care provided to the characteristics of physicians, other clinic staff, and the clinic environment. There was relatively restricted variability in ratings of importance across topics, features, and ideas, underscoring their shared contributions to the meaning culturally safe primary care. The purpose of this study was not to evaluate the accuracy of peoples’ past experiences of stigma or safe/unsafe health care, but to relay what people perceive to be key aspects of safety in health care, in the context of a cultural identity that is highly marginalized and even criminalized. Planning and delivering culturally safe health care requires that providers and policy makers reflect on their own power and positioning, and how forms of structural disadvantage and violence are reproduced in health care settings and policies. It is worth nothing that interactions with health care providers may be experienced by patients as unsafe despite the intentions of providers or their impressions of the encounter and quality of care [34]. Within the eight themes that comprise this model of cultural safety, there are numerous concrete suggestions for providers who are looking to explore ways to improve access and service delivery for people who use substances. Findings offer various potential accommodations that may be tested in future research for their effectiveness in improving access and outcomes among people who use substances. As part of our knowledge exchange strategy, we have translated findings into an anti-stigma workshop for primary care providers, and have created a post card summarizing study findings, for use by community members in advocating for their own care.

In terms of serving as a driver for systems change, it is relevant that our findings are strongly consistent with prior studies describing the key components of equity-oriented primary care, which include being responsive to inequities and the social determinants of health, being trauma-and violence-informed, and being tailored to peoples’ social and cultural contexts [7, 45]. Likewise, a role for advocacy is aligned with recommendations to find places to disrupt. A number of specific strategies identified in prior work to enhance these components of equity-oriented primary care were echoed by participants in our study (e.g., actively working to counter oppression and promote equity, allowing for more time in appointments, building a rapport, allowing for flexibility in scheduling, tailoring care to the individual and the contexts in which they live) [7]. The consistency across studies speaks to the credibility of these findings.

Also consistent with prior findings are the experiences of stigma, labeling, and stereotyping that people who use substances reported in describing their interactions with physicians and other clinic staff, including receptionists and office assistants [7]. While participants relayed past experiences with both culturally safe and unsafe care, it is the case that the majority of experiences were negative. Participants were mindful of their own role in contributing to these negative experiences, and many expressed some sympathy with physicians on the difficulty of their profession. It is clear that participants were highly attuned to potentially dismissive or stigmatizing attitudes and behaviors of physicians and clinic staff, and that past negative experiences affected the ways in which they approached health care interactions. Similar processes have been noted by other socially marginalized groups, including people who identify as Indigenous [34, 46], and speak to the need for care providers to be mindful of past traumas and other experiences of their patients. Many of the elements of this model of culturally safe primary care align with broader definitions of health care quality (for instance, receiving care that aligns with professional standards or having confidentiality maintained). That is, the elements of safe care described here are not unique to people who use substances, but reflect more widely shared expectations of primary care. The fact that such issues were raised explicitly speaks to the sizable amount of work that is required to achieve equity-oriented primary care for people who use substances.

Findings suggest that interventions designed to support the development of positive relationships between primary care providers and people who use substances are warranted. Although more work is needed to establish effective ways to achieve this, there is some evidence to support the effectiveness of interventions in which people with lived experience of substance use engage with and educate health care providers in improving care relationships [47, 48]. In particular, there is a critical need to attend to the building of trust in relationships between individuals and with groups in health care [21]. Others have moved beyond the micro-level of individual providers to design organizational-level interventions that attend to the ways in local and system contexts affect equity in primary care (both in terms of front-end access and processes of service delivery) [45]. The theoretical model guiding such efforts posits that, by attending to and tailoring activities to fit the organizational contexts in which care is delivered, interventions will be more successful at fostering organizational structures and policies that support equity-oriented care and, in turn, more equitable health outcomes in the population.

We also recognize that supportive structures and policies need to be in place in order for physicians and other primary care providers to be able to deliver culturally safe primary care, and that some of the accommodations and strategies outlined in this study will require significant systems change (e.g., changes to funding and remuneration, educational curricula). In Canada, primary care is covered by universal provincial health insurance; although challenges related to keeping up-to-date identification cards and maintaining active registration can also affect coverage among members of marginalized groups (e.g., people who are homeless) [18]. This accounts in part for statements reflecting a fear of being turned-away or not being covered by insurance, despite universal health care coverage. Spending adequate time with patients is also a major challenge for primary care physicians practicing in Canada, many of whom are paid primarily through a fee-for-service structure associated with the universal health insurance plan. Successful implementation of culturally safe primary care requires critical reflection not only by health care providers, but also by decision-makers and health planners. In this sense, the present study provides evidence that may be useful to physicians, other providers, and their representative organizations as a tool for advocacy efforts. As noted earlier, cultural safety provides a potentially useful lens through which health care providers can work towards equity and social justice within their professions [37, 38].

Part of the value of the concept of cultural safety lies in the fact that it is defined by the care recipients, and therefore requires meaningful involvement of patients in defining and interpreting what is considered to be safe care. The stigma of substance use, particularly illicit drug use, is one way through which people who use substances are excluded from social structures where decisions are made around the design and implementation of health services [16, 49–51]. When people are excluded from decision-making processes (whether for their own health care or for the design and implementation of services), stigma can be unintentionally reproduced and opportunities to provide health care are missed. Capacity-building among people who use substances is critical for facilitating the collective efficacy and political voice necessary to raise awareness and address stigma in health care [52, 53]. As a precedent, many innovations in harm reduction services and policies have been driven by people who use illicit drugs with the goal of promoting the health and safety of their peers [54–58].

We made efforts to maximize the credibility of our findings; for instance, by giving participants the opportunity at each data collection point to review the statements and clusters and make any required changes to better reflect their perceptions of cultural safety. We were also successful at recruiting a diverse sample of people who use substances through the contributions and work of the community researchers. That said, our study is limited by a number of factors. Not all of those who were recruited could be located for the second and third rounds of focus groups, such that the sample differed slightly across time points. While the sample includes representation from Indigenous peoples including women, and their perspectives informed study conclusions, these findings are not explicit to their needs and preferences. We did not obtain representation of those with diverse gender identities or sexual orientations, or youth who use substances. Further work is needed to explore cultural safety in these populations. A small number of participants experienced difficulties completing the task of sorting the statements. In these cases, a member of the research team provided assistance, by reading the statements out loud and asking for direction on what piles to create and where to put each statement. The study was conducted in one community and, therefore, may reflect how stigma and primary care are experienced locally. Relatedly, it is needs to be acknowledged that this study was conducted within the context of an escalating public health emergency related to illicit drug overdoses. This context is shaped by the fact that harm reduction is official provincial policy in British Columbia, Canada, and that public campaigns to reduce stigma were ongoing during the period of data collection as part of strategy to reduce overdoses.

## Conclusions

Cultural safety is recognized as a key aspect of health care, particularly as it relates to Indigenous peoples [46]. This study makes a valuable contribution to understandings of culturally safe primary care for people who are often socially marginalized and stigmatized by their substance use. A major strength of the study is the inclusion of community researchers who work in both harm reduction and abstinence-based services. Findings highlight the importance placed on non-judgmental care that acknowledges peoples’ past experiences of stigma in health care settings, as well as socioeconomic and structural disadvantages and how these can affect health care experiences and outcomes. They offer numerous potential opportunities for accommodating and reducing the anxiety that people can feel in going to the doctor, through interpersonal exchanges, environmental features of the clinic, and others. Future work is required to develop ways to implement these findings into clinic practices and processes and test their effectiveness in promoting equity-oriented primary care for people who use substances.

## Data Availability

The datasets used and/or analysed during the current study are available from the corresponding author on reasonable request.

## Abbreviation

HCV: Hepatitis C virus
